# 2570. The intraoperative pharmacokinetics of ampicillin and sulbactam and the effect of massive bleeding in recipients of living donor liver transplantation

**DOI:** 10.1093/ofid/ofad500.2187

**Published:** 2023-11-27

**Authors:** Yuji Wakimoto, Koh Okamoto, Takehito Yamamoto, Nobuhisa Akamatsu, Taro Kariya, Yoko Hoshino, Sohei Harada, Hideki Hashimoto, Daisuke Jubishi, Ryo Yamaguchi, Takehiro Tanaka, Junichi Kaneko, Shu Okugawa, Kanji Uchida, Tappei Takada, Kiyoshi Hasegawa, Takeya Tsutsumi

**Affiliations:** The University of Tokyo Hospital, Bunkyo, Tokyo, Japan; The University of Tokyo Hospital, Bunkyo, Tokyo, Japan; The University of Tokyo Hospital, Bunkyo, Tokyo, Japan; The University of Tokyo Hospital, Bunkyo, Tokyo, Japan; The University of Tokyo Hospital, Bunkyo, Tokyo, Japan; The University of Tokyo Hospital, Bunkyo, Tokyo, Japan; The University of Tokyo Hospital, Bunkyo, Tokyo, Japan; The University of Tokyo Hospital, Bunkyo, Tokyo, Japan; The University of Tokyo Hospital, Bunkyo, Tokyo, Japan; The University of Tokyo Hospital, Bunkyo, Tokyo, Japan; The University of Tokyo Hospital, Bunkyo, Tokyo, Japan; The University of Tokyo Hospital, Bunkyo, Tokyo, Japan; The University of Tokyo Hospital, Bunkyo, Tokyo, Japan; The University of Tokyo Hospital, Bunkyo, Tokyo, Japan; The University of Tokyo Hospital, Bunkyo, Tokyo, Japan; The University of Tokyo Hospital, Bunkyo, Tokyo, Japan; The University of Tokyo Hospital, Bunkyo, Tokyo, Japan

## Abstract

**Background:**

The current guidelines recommend redosing of prophylactic intraoperative antimicrobials based on their half-life or in case of bleeding greater than 1,500 mL. However, data to support this practice is scarce. Orthotopic liver transplantation is often accompanied by massive bleeding. Therefore, we aimed to characterize the intraoperative pharmacokinetics of the prophylactic antimicrobial to analyze the pharmacokinetic parameters in recipients of living-donor liver transplantation (LDLT).

**Methods:**

The recipients of LDLT who were 20 years or older without allergy to any beta-lactams were included. All participants received intravenous ampicillin-sulbactam (ABPC-SBT) 2-1 gram one hour before and every three hours during surgery over 30 minutes. The amount of blood loss and relevant parameters were recorded. Blood samples were collected every hour during surgery. The total plasma concentrations of ABPC and SBT were determined using a liquid chromatography-tandem mass spectrometry. Noncompartmental analysis was applied to calculate the clearances of ampicillin and sulbactam. The probability of target attainment (PTA) was determined based on the breakpoints proposed by The Clinical Laboratory Standards Institute assuming that the protein binding rate of ABPC and SBT were 28% and 38%, respectively.

**Results:**

Twenty recipients were included (Table). The median age, weight, and amount of blood loss of the recipients were 52 years, 62.1 kg, and 11,158 mL, respectively. A median of calculated creatinine clearance were 99.6 mL/min. The total concentrations of ABPC and SBT in plasma and the amount of blood loss are shown (Figure 1, 2). Twelve participants (60%) achieved the PTA of 100% in both ABPC and SBT during surgery. The model-independent clearances of ABPC and SBT were 80.3 and 77.2 mL/min, respectively. Although bleeding greater than 20,000mL tended to increases the clearances of ABPC and SBT, the overall time above minimum inhibitory concentration of common pathogens was at acceptable level during surgery.

The pharmacokinetics of ampicillin in the recipients in the living donor liver transplantation.

**Figure:**
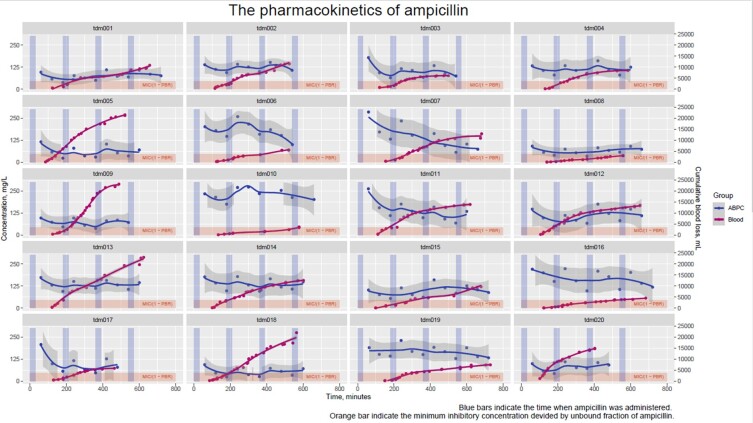

The pharmacokinetics of sulbactam in the recipients in the living donor liver transplantation.

**Figure:**
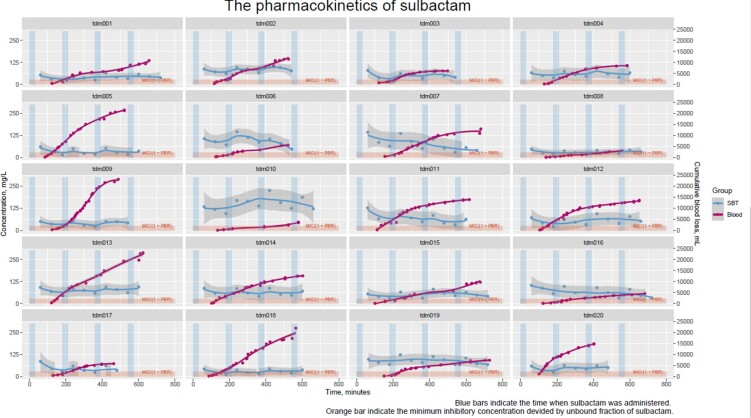

**Conclusion:**

Intraoperative redosing of ABPC-SBT every three hours achieved the PTA of 100% in 60% of the LDLT recipients despite intraoperative massive bleeding.

**Disclosures:**

**All Authors**: No reported disclosures

**Table.** Characteristics of 20 living-donor liver transplantation recipients.

	N (%) or median (interquartile range)
Age, years	52.0 (44.8-56.0)
Female sex	11 (55%)
Body weight, kg	62.1 (53.5-74.5)
Body mass index, kg/m^2^	22.5 (19.8-25.2)
Underlying liver diseases	
Alcoholic liver disease	8 (40 %)
Nonalcoholic steatohepatitis	4 (20 %)
Autoimmune hepatitis	2 (10 %)
Primary sclerosing cholangitis	2 (10 %)
Primary biliary cholangitis	1 (5 %)
Congenital biliary atresia	1 (5 %)
Idiopathic portal hypertension	1 (5 %)
Multiple cause	1 (5 %) *
MELD score	18.5 (14.5-23.5)
Ratio of graft weight to recipient standard liver volume	0.46 (0.41-0.50)
Renal replacement therapy before transplantation	2 (10 %)
Serum creatinine concentration, mg/dL	0.72 (0.56-1.03)
Calculated creatinine clearance (Cockcroft-Gault equation), mL/min	99.57 (88.6-107.3) **
Serum albumin concentration, g/dL	2.90 (2.65-3.15)
Total operation time, hours	9.6 (8.5-10.2)
Total blood loss, mL	11,158 (6,032-14,125)
Total intraoperative infusion and transfusion, mL	16,900 (11,248-21,675)
Crystalloid, mL	3175 (2,238-3,900)
Colloid, mL	2375 (1,500-3,938)
Red blood cell, mL	4320 (2,340-5,640)
Fresh frozen plasma, mL	6,660 (3,600-8,400)
Platelet component, mL	800 (400-1,000)

*Chronic hepatitis B, chronic hepatitis D, and autoimmune hepatitis

** Recipients on renal replacement therapy were excluded.

